# Supplementary material for: Inhibition of epithelial cell YAP-TEAD/LOX signaling attenuates pulmonary fibrosis in preclinical models
Source: Nat Commun. 2025 Aug 2;16:7099. doi: 10.1038/s41467-025-61795-x (PMC12318044; doi:10.1038/s41467-025-61795-x)
Supplement: Supplementary file 1 — Supplementary Information [file 41467_2025_61795_MOESM1_ESM.pdf]

Inhibition of epithelial cell YAP-TEAD/LOX signaling attenuates pulmonary fibrosis

Author List: Darcy Elizabeth Wagner*[1-8], Hani N Alsafadi[1-5], Nilay Mitash[9,10], Aurelien Justet[11,12], Qianjiang Hu[9,10], Ricardo Pineda[9,10], Claudia Staab-Weijnitz[13,14], Martina Korfei[15,16], Nika Gvazava[1-4], Kristin Wannemo[9], Ugochi Onwuka[9], Molly Mozurak[9], Adriana Estrada-Bernal[9], Juan Cala-Garcia[17,18], Kathrin Mutze[5], Rita Costa[5], Deniz Bölükbas[1-3], Jon Stegmayr[1-4], Wioletta Skronska-Wasek[5], Stephan Klee[5], Chiharu Ota[5], Hoeke A Baarsma[5], Jingtao Wang[6], John Sembrat[9], Anne Hilgendorff[13], Jun Ding[6], Andreas Günther[15,16], Rachel Chambers[19], Ivan Rosas[18], Stijn de Langhe[20], Naftali Kaminski[11], Mareike Lehmannn[5,13, 21, 22] Oliver Eickelberg[9], Melanie Königshoff[5,9,10]*

Affiliations:

[1] Lund Stem Cell Center, Faculty of Medicine, Lund University, Lund, Sweden

[2] Lung Bioengineering and Regeneration, Department of Experimental Medical Sciences, Faculty of Medicine Lund University, Lund, Sweden

[3] Wallenberg Center for Molecular Medicine, Faculty of Medicine, Lund University, Lund, Sweden

[4] NanoLund, Lund University, Lund, Sweden

[5] Lung Repair and Regeneration Research Unit, Helmholtz Zentrum München, Member of the German Center for Lung Research (DZL), Munich, Germany

[6] Meakins-Christie Laboratories, Research-Institute of the McGill University Hospital, Montreal, Canada

[7] Department of Medicine, McGill University, Montreal, Canada

[8] Department of Biomedical Engineering, McGill University, Montreal, Canada

[9] Division of Pulmonary, Allergy, Critical Care, and Sleep Medicine, Department of Medicine University of Pittsburgh, Pittsburgh, USA

[10] Geriatric Research Education and Clinical Center (GRECC) at the VA Pittsburgh Healthcare System, Pittsburgh, Pennsylvania.

[11] Pulmonary, Critical Care and Sleep Medicine, Yale School of Medicine, New Haven, CT, USA

[12] Department of Pulmonary Medicine, Interstitial Lung Disease Center, University Hospital of Caen UNICAEN, Caen Normandie, CNRS, Normandie University, ISTCT, UMR6030, GIP Cyceron, F-14000 Caen, France

[13] Institute for Lung Health and Immunity, Helmholtz Zentrum München and University Hospital of the Ludwig Maximilians Universität, Member of the German Center for Lung Research (DZL), Munich, Germany

[14] University of Colorado, Anschutz Medical Campus, School of Medicine, Department of Pediatrics, and Division of Pulmonary Sciences and Sleep Medicine, Aurora, CO, USA

[15] Dept of Internal Medicine, Justus-Liebig-Universität Giessen, Giessen, Germany

[16] Universities of Giessen and Marburg Lung Center (UGMLC), Member of the German Center for Lung Research (DZL), Giessen, Germany

[17] Department of Internal Medicine. Yale University, New Haven, CT

[18] Section of Pulmonary, Critical Care, and Sleep Medicine, Department of Medicine, Baylor College of Medicine, Houston, TX, USA

[19] Centre for Inflammation and Tissue Repair, UCL Respiratory, University College London, London, UK

[20] Department of Medicine, Division of Pulmonary and Critical Medicine, Mayo Clinic, Rochester, United States

[21] Institute for Lung Research, Philipps-University Marburg, Marburg, Germany

[22] Institute for Lung Health, German Center for Lung Research (DZL), Giessen, Germany

***Co-corresponding Authors. Email:** koenigshoffm@upmc.edu and darcy.wagner@mcgill.ca

Keywords: Fibrosis, respiratory disorder, epithelial cell reprogramming, YAP/TAZ, Hippo, extracellular matrix, LOX

Supplementary Materials

List of Supplementary Figures and Tables

Figure S1. Representative immunohistochemical staining of YAP, TAZ, epithelial, and mesenchymal markers on tissue sections for healthy tissue, and IPF tissues (moderate fibrosis and full fibrosis)

Figure S2. Experimental overview for in vivo verteporfin treatment and monitoring.

Figure S3. Transcriptional changes in the major fibrillar collagen chains *Col1a1* and *Col3a1* following verteporfin treatment in healthy (PBS) and fibrotic (bleomycin-treated) mice.

Figure S4. mRNA expression of *Yap1*, *Wwtr1* (Taz), and *Ctgf (Ccn2)* in distal epithelial cells isolated from PBS and Bleomycin treated mice after knockdown of Yap/Taz with siRNA.

Figure S5. Additional images and no primary controls corresponding to Figure 3H.

Figure S6. Single channel images and digital zoom insets (2x) for a and b, corresponding to Figure 3H

Figure S7. *Loxl1* levels in bleomycin-induced pulmonary fibrosis with or without every other day i.p. verteporfin administration as described in Figure S2

Figure S8. Knockdown of Lox in primary murine AT2 cells does not alter the AT2 phenotype or induce senescence.

Figure S9. TWOMBLI analysis of collagen hydrogels shown in Figure 5B formed in the presence of the secretome from normal or fibrotic AT2 cells treated with or without verteporfin.

Figure S10. SEM of collagen gels produced in cell-free collagen formation assay with supernatants of normal and fibrotic pmAT2 cells with siYT

Figure S11. Single Uniform Manifold Approximation and Projection (UMAP) representation of snRNAseq from Precision Cut Lung slices

Figure S12. Effects of FC and FC+VP on major cell types.

Figure S13. Dot plots corresponding to snRNA-seq data for cluster assignment.

Figures S14. Dot plots of LOX family members corresponding to Figure 6H.

Figure S15. *Lox, Loxl1* and *Loxl2* expression in all major cell types.

Figure S16. Overview of the most abundantly expressed MMPs in epithelial cells in PCLS treated with FC and verteporfin.

Figure S17. Secreted LOX expression in supernatants collected from 4mm diameter hPCLS treated with CC/FC or verteporfin (FC).

Figure S18. (A-F) Gene expression of *FN1*, and *COL1A1* in PCLS treated with the fibrosis cocktail and VP, Pirfenidone, or Nintedanib

Table S1: Hippo pathway gene list used for Principal component analysis (PCA) (Correaponding to Figure 1D.

Table S2: REACTOME analysis for genes downregulated following siYap/Taz in fibrotic alveolar cells.

Table S3: Identifiable Peaks Identified in Paraffin Embedded Lung Tissue Slices Corresponding to IR Bands Found in the Literature

Table S4. Human primer sets used for RT-qPCR (Corresponding to Figure 7A and Figure 7C)

Table S5. Mouse primer sets used for RT-qPCR (Corresponding to Figure 2C, Figure 3D, Figure 4A, and Figure 4D)

Table S6. Antibodies used for immunohistochemistry (IHC), immunofluorescence (IF) and western blot (WB) (Corresponding to Figure 1B, Figure 1C, Figure 2A, Figure 3A, Figure 3E, Figure 4B, Figure 4C, Figure 4E, Figure 5C, and Figure 7B)

Supplementary Figures

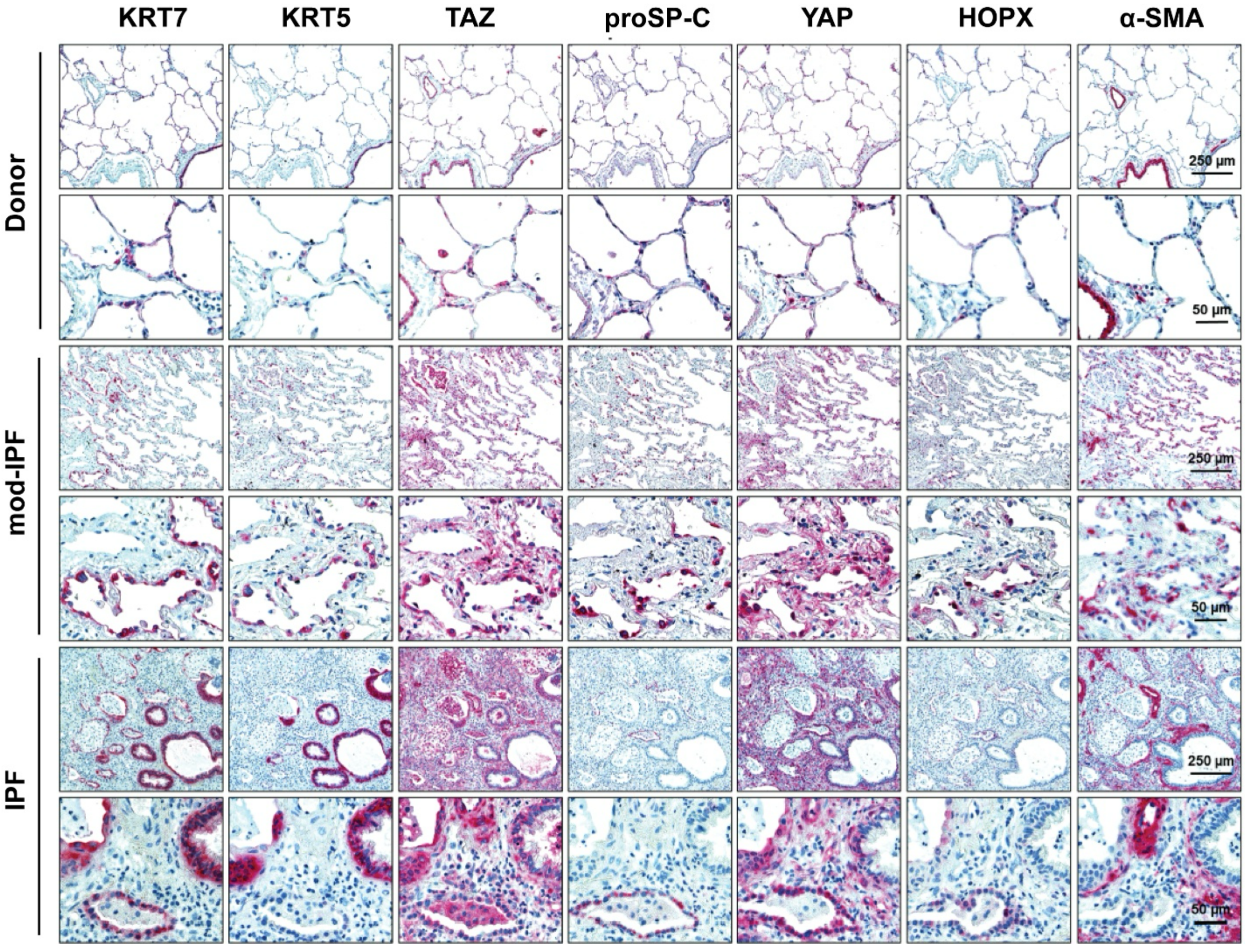

Figure S1. Representative immunohistochemical staining of YAP, TAZ, epithelial, and mesenchymal markers on tissue sections for healthy tissue, and IPF tissues (moderate fibrosis and full fibrosis), n=6 healthy, n=6 mod-IPF, n=13 IPF. Source data are deposited at S-BIAD1520.

A

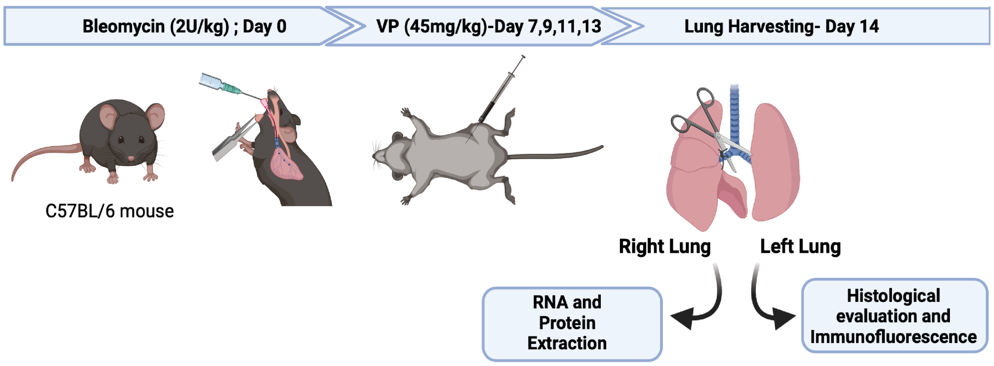

B

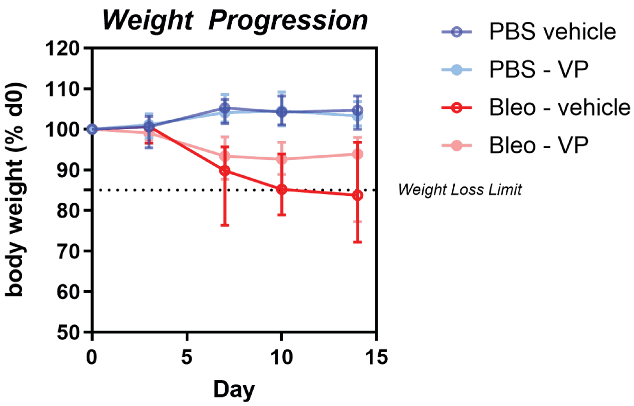

Figure S2. Experimental overview for *in vivo* verteporfin treatment and monitoring. A) Schematic for intratracheal bleomycin administration using a microsprayer following intubation to C57BL/6 mouse to induce fibrosis and successive VP treatment administered intraperitoneally starting at day 7 for every 48h until day 14. Animals were randomized into groups prior to the onset of all experiments (i.e. prior to day 0). Created in BioRender. Wagner, D. (2025) https://BioRender.com/nmwzx11 B) Weight loss curves at day 0, 3, 7, 10, and 14 displaying median and 95% confidence interval. Dotted line at 85% shows maximum allowed weight loss per local ethical regulations. No statistical differences were observed between the two PBS groups at any time point or at day 7, prior to VP administration, between VP or vehicle administration. PBS groups were statistically different to both bleomycin groups at day 7, 10 and 14, $p<0.05$ considered significant. Statistical testing was performed using 2-Way ANOVA with Bonferonni's multiple comparison test. Source data and statistics for Panel B are provided as a Source Data file.

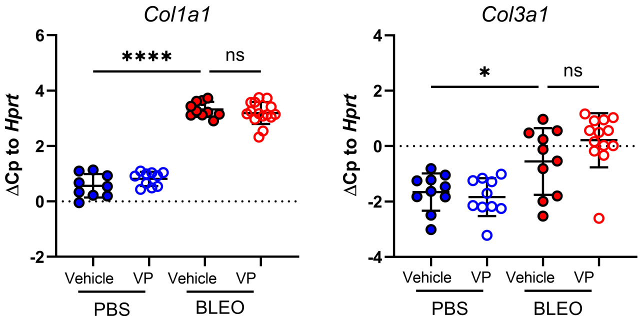

Figure S3. Transcriptional changes in the major fibrillar collagen chains *Col1a1* and *Col3a1* following verteporfin treatment in healthy (PBS) and fibrotic (bleomycin-treated) mice. One-way ANOVA with Holm-Šídák's multiple comparisons test and *adjusted p<0.05 considered significant. *Col1a1*: PBS-veh vs. PBS-VP (p=0.2429); PBS-veh vs. Bleo-veh (p<0.0001); Bleo-veh vs. Bleo-VP (p=0.3677). *Col3a1*: PBS-veh vs. PBS-VP (p=0.6581); PBS-veh vs. Bleo-veh (p=0.0306); Bleo-veh vs. Bleo-VP (p=0.1045). Mean ± s.d. Source data are provided as a Source Data file.

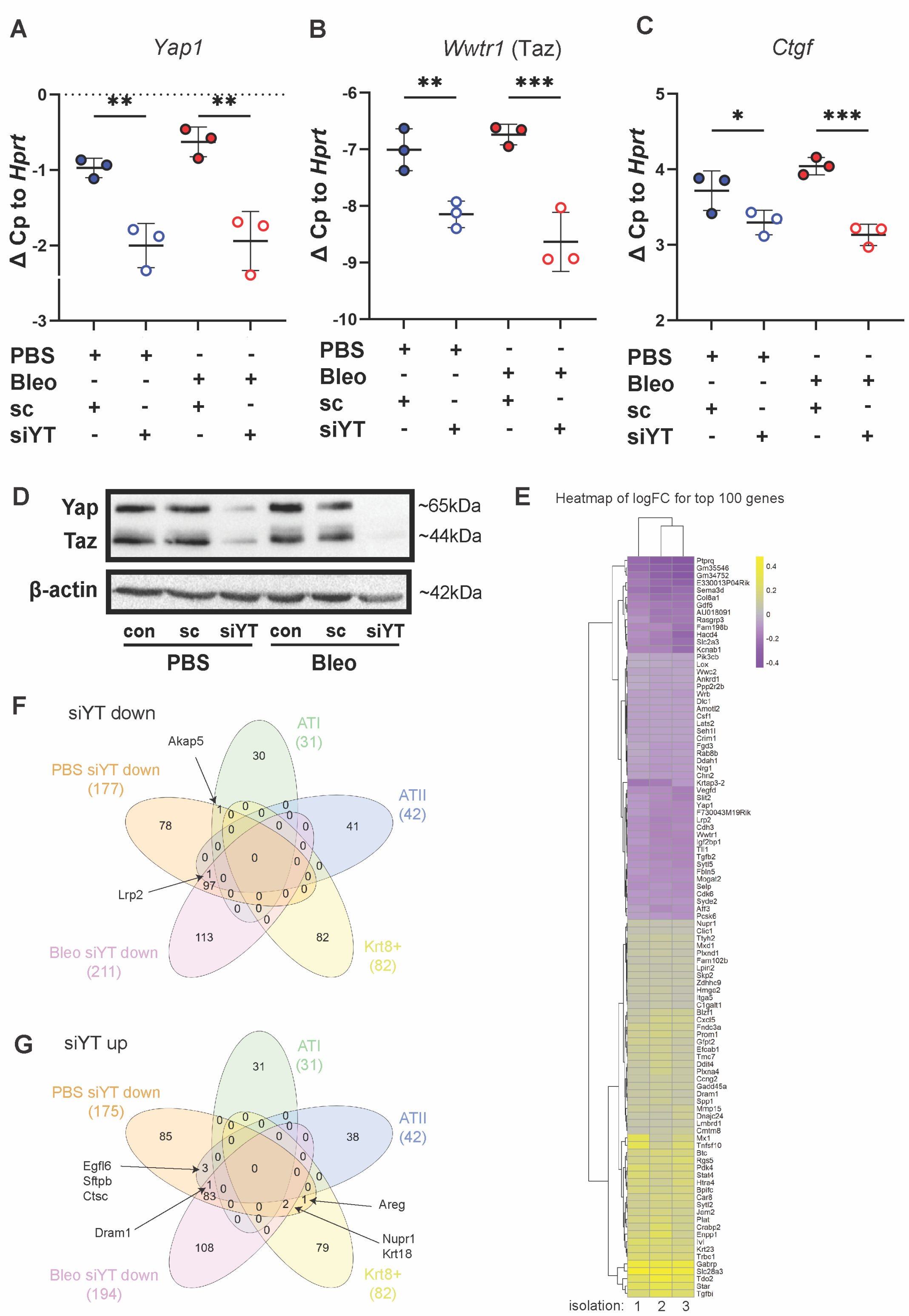

Figure S4. (**A-C**) mRNA expression of *Yap1*, *Wwtr1* (Taz), and *Ctgf (Ccn2)* in distal epithelial cells isolated from PBS and Bleomycin treated mice after knockdown of Yap/Taz with siRNA. *p<0.05 considered significant with one-way ANOVA with Holm-Šídák's multiple comparisons test. Mean ± s.d. *Yap1:* PBS-sc vs. PBS-siYT (p=0.0033), PBS-sc vs. Bleo-sc (p=0.1574), Bleo-sc vs. Bleo-siYT (p=0.0010); *Wwtr1*: PBS-sc vs. PBS-siYT (p=0.0083), PBS-

sc vs. Bleo-sc (p=0.3792), Bleo-sc vs. Bleo-siYT (p=0.0005); *Ctgf*: PBS-sc vs. PBS-siYT (p=0.409), PBS-sc vs. Bleo-sc (p=0.575), Bleo-sc vs. Bleo-siYT (p=0.0008); Mean +/- s.d. (**D**) Protein expression of Yap1, and Taz (*Wwtr1*) in distal epithelial cells isolated from PBS and Bleomycin treated mice after knockdown of Yap/Taz. Beta-actin as loading control. **(E)** Top 100 genes downregulated after knockdown of Yap/Taz in distal epithelial cells isolated from PBS and Bleomycin treated mice. **(F,G)** Overlap of genes significantly downregulated (**F**) or upregulated (**G**) with adjusted p.value <0.1 for distal epithelial cells isolated from PBS and Bleomycin treated mice after knockdown of Yap/Taz and compared to marker gene lists identified previously[1] from GSE141259 using the FindAllMarkers() in Seurat for genes with logFC greater than 0.5. Venn diagrams generated with Interactivenn[2]. Source Data for Panel A-G is deposited as Source Data file and H is deposited at E-MTAB-14643.

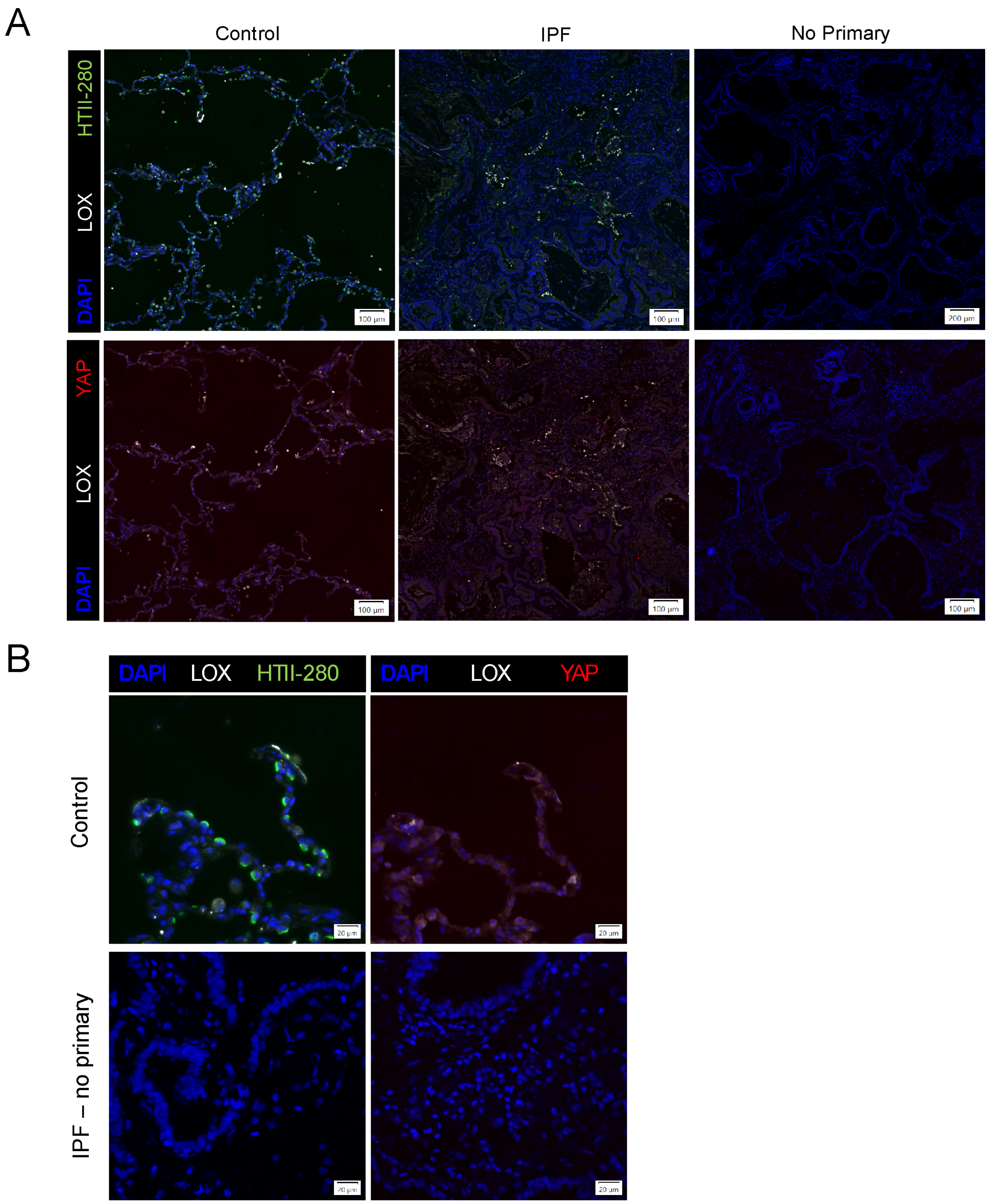

Figure S5. Additional images and no primary controls corresponding to Figure 3H. A) Low magnification (4x digital zoom) of control, IPF and no primary for LOX, HTII-280 and YAP. To avoid the known tissue autofluorescence encountered in the FITC channel, secondary antibodies corresponding to Cy5 (LOX) and Cy7 (HTII-280) were used on a parallel section of an IPF lung which has treated identically to all other samples but was not incubated with a primary antibody. **B)** Donor controls (20x digital zoom) and no primary controls (IPF tissue) for images in Figure 3H. All original images taken with a 20x objective and images were exported using the Olympus OlyVia 3.1 Software. Source data for all panels is deposited at S-BIAD1520.

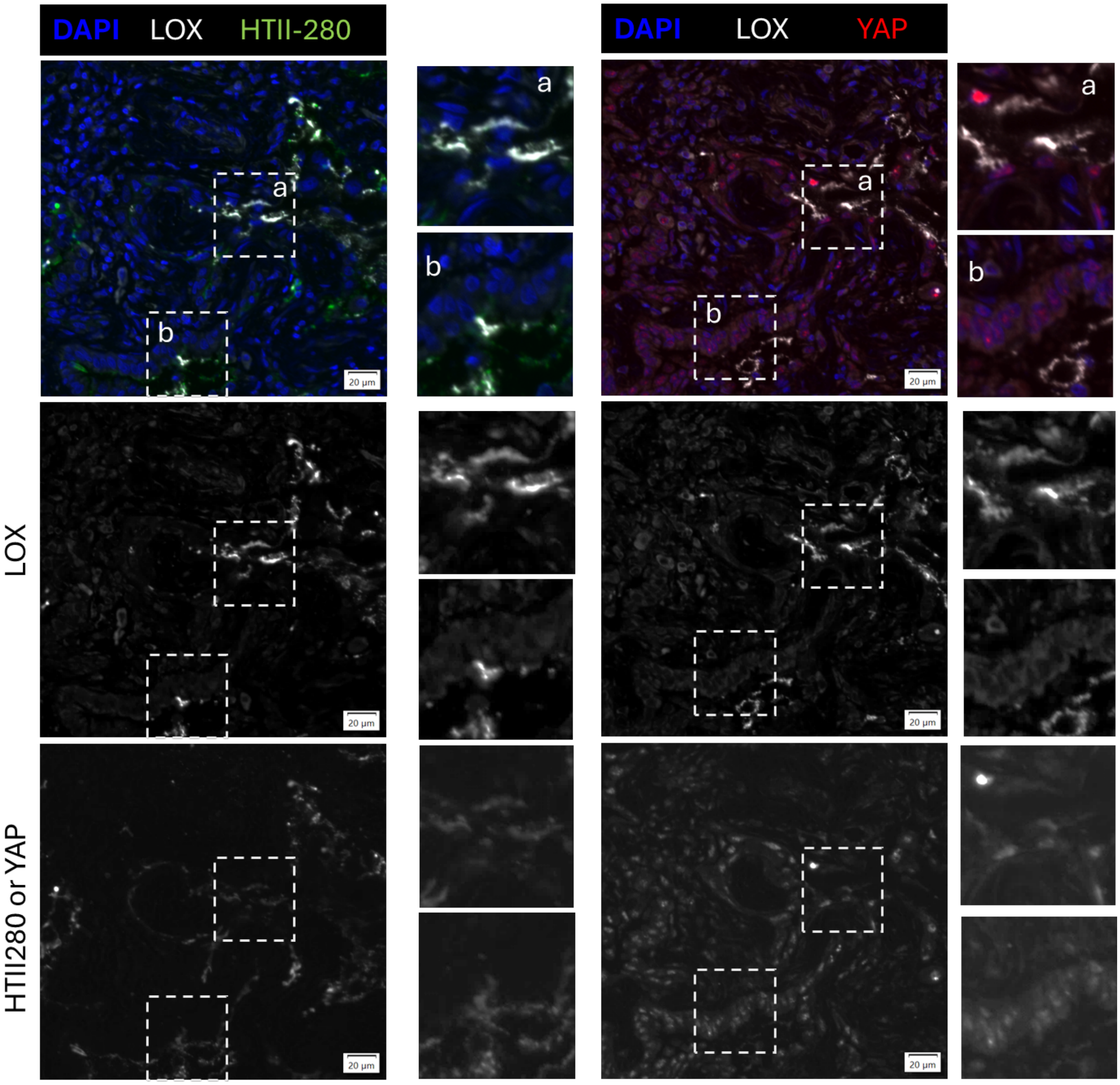

Figure S6. Single channel images and digital zoom insets (2x) for a and b, corresponding to Figure 3H. Images from 3H are repeated here in the top row. All original images taken with a 20x objective and images were exported using the Olympus OlyVia 3.1 Software with a (20x) digital zoom. Brightness and contrast adjusted for insets uniformly across the entire image dataset. Source data for all panels is deposited at S-BIAD1520.

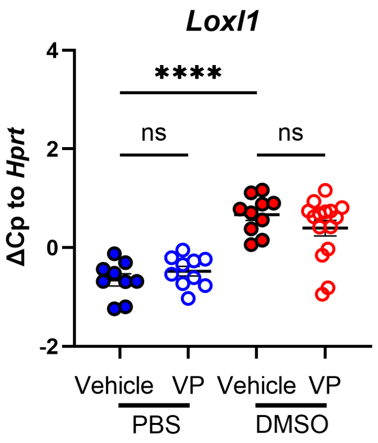

Figure S7. *Loxl1* levels in bleomycin-induced pulmonary fibrosis with or without every other day i.p. verteporfin administration as described in Figure S2. *adjusted p<0.05 considered significant with one-way ANOVA with with Holm-Šídák's multiple comparisons test. PBS-veh vs. PBS-VP (p=0.4157), PBS-veh vs. Bleo-veh (p<0.0001), Bleo-veh vs. Bleo-VP (p=0.2836); Mean ± s.d. Source data is provided as a Source Data file.

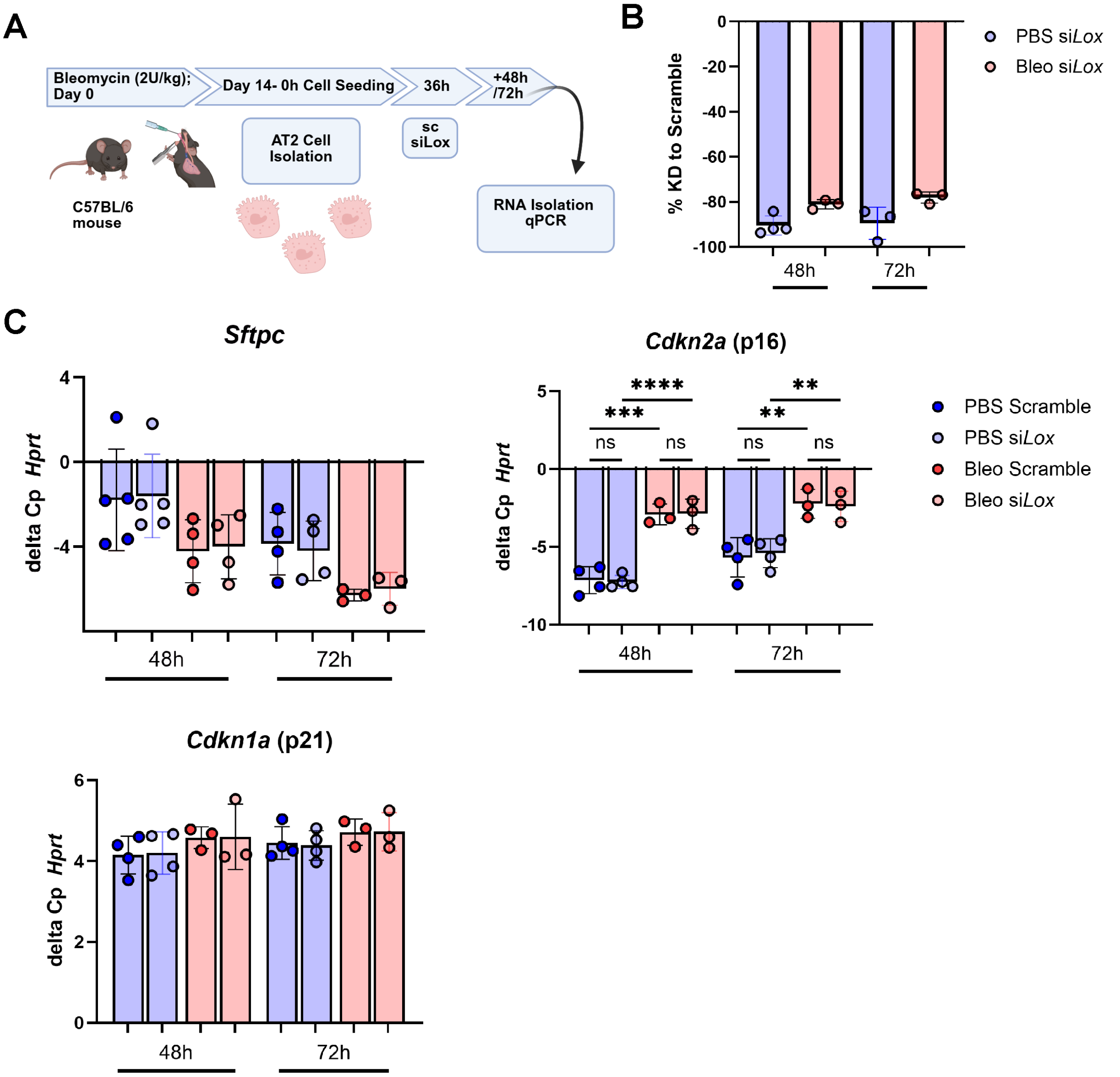

Figure S8. Knockdown of *Lox* in primary murine AT2 cells does not alter the AT2 phenotype or induce senescence. A) Schematic overview Created in BioRender. Wagner, D. (2025) https://BioRender.com/nmwzx11 n=3 or 4 independent pmAT2 isolations, as indicated in each graph. B) Knockdown efficiency (KD). C) Phenotypic (*Sftpc*) and senescent markers (*Cdkn1a* and *Cdkn2a*) Mean ± s.d. *p<0.05 considered statistically significant. One-way ANOVA with with Holm-Šídák's multiple comparisons test. No significance detected in Panel C for *Sftpc* or *Cdkn1a*. *Cdkn2a:* 48 hours) PBS-sc vs PBS-siLox (p=0.9955); PBS-sc vs. Bleo-sc (p=0.0001); Bleo-sc vs. Bleo-siLox (p=0.9955); PBS-siLox vs. Bleo-siLox (p<0.0001) and 72 hours) PBS-sc vs PBS-siLox (p=0.9899); PBS-sc vs. Bleo-sc (p=0.0016); Bleo-sc vs. Bleo-siLox (p=0.9955); PBS-siLox vs. Bleo-siLox (p=0.0055). Source data and all statistical comparisons are provided as a Source Data file.

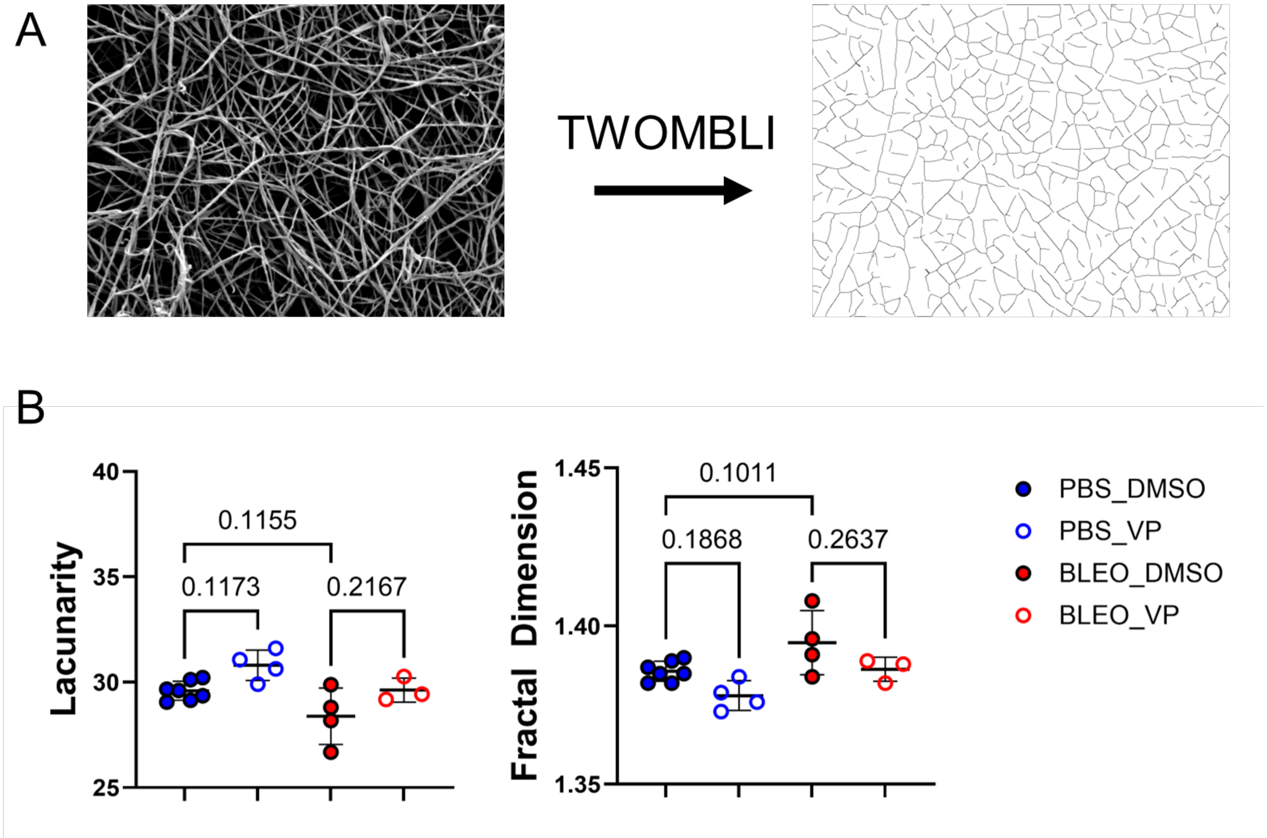

Figure S9. TWOMBLI[3] analysis of collagen hydrogels shown in Figure 5B formed in the presence of the secretome from normal or fibrotic AT2 cells treated with or without verteporfin. A) Representative transformation of SEM image after running TWOMBLI. B) Lacunarity is a measure of how the ECM fills the space (i.e. the number and size of gaps in the matrix). Larger values indicate larger space in the matrix pattern. Fractal dimension is a measure of dimension and similarity. It spans between 1 (a thin line) and 2 (filled square) with lower fractal dimensions present in ECM with more open space. Line widths of 30 and minimum branch length of 15 were used with maximum contrast adjusted so that only SEM fibers on the topmost layer were analysed. n=3-7 are indicated on the figure; statistical significance was assessed using one-way ANOVA with Tukey post-test with p-values labeled on the graph and $p<0.05$ considered significant. Mean ± s.d. Source data for Panel B is provided as a Source Data file and raw images used for analysis are deposited at S-BIAD1520.

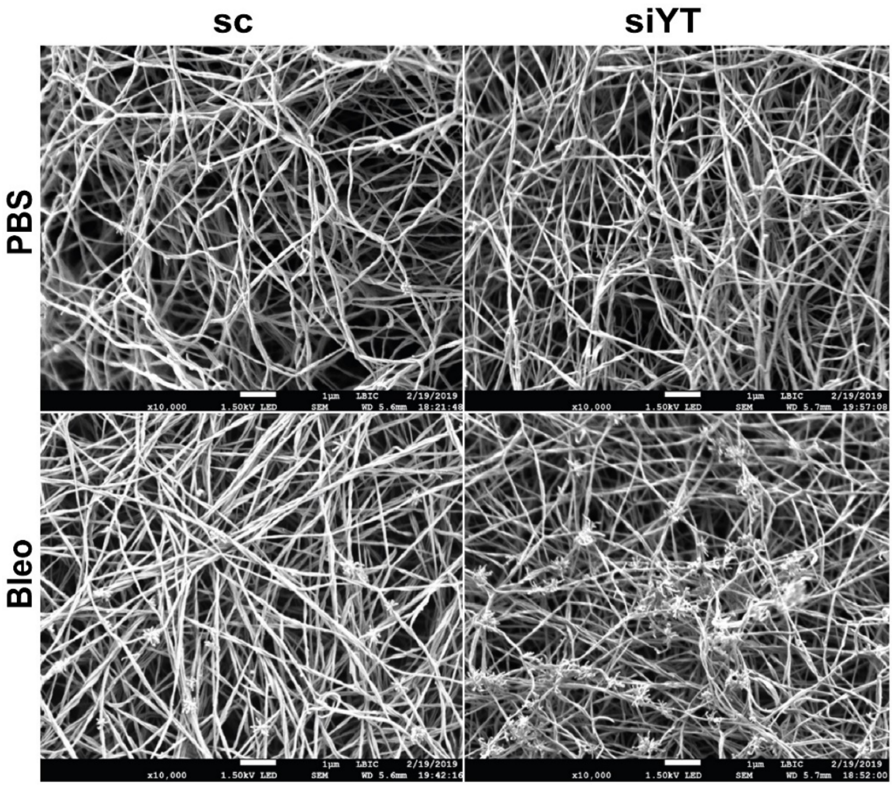

Figure S10. SEM of collagen gels produced in cell-free collagen formation assay with supernatants of normal and fibrotic pmAT2 cells with siYT, n=3 individual isolations from single or pooled mice. Source data for these and additional n's is deposited at S-BIAD1520.

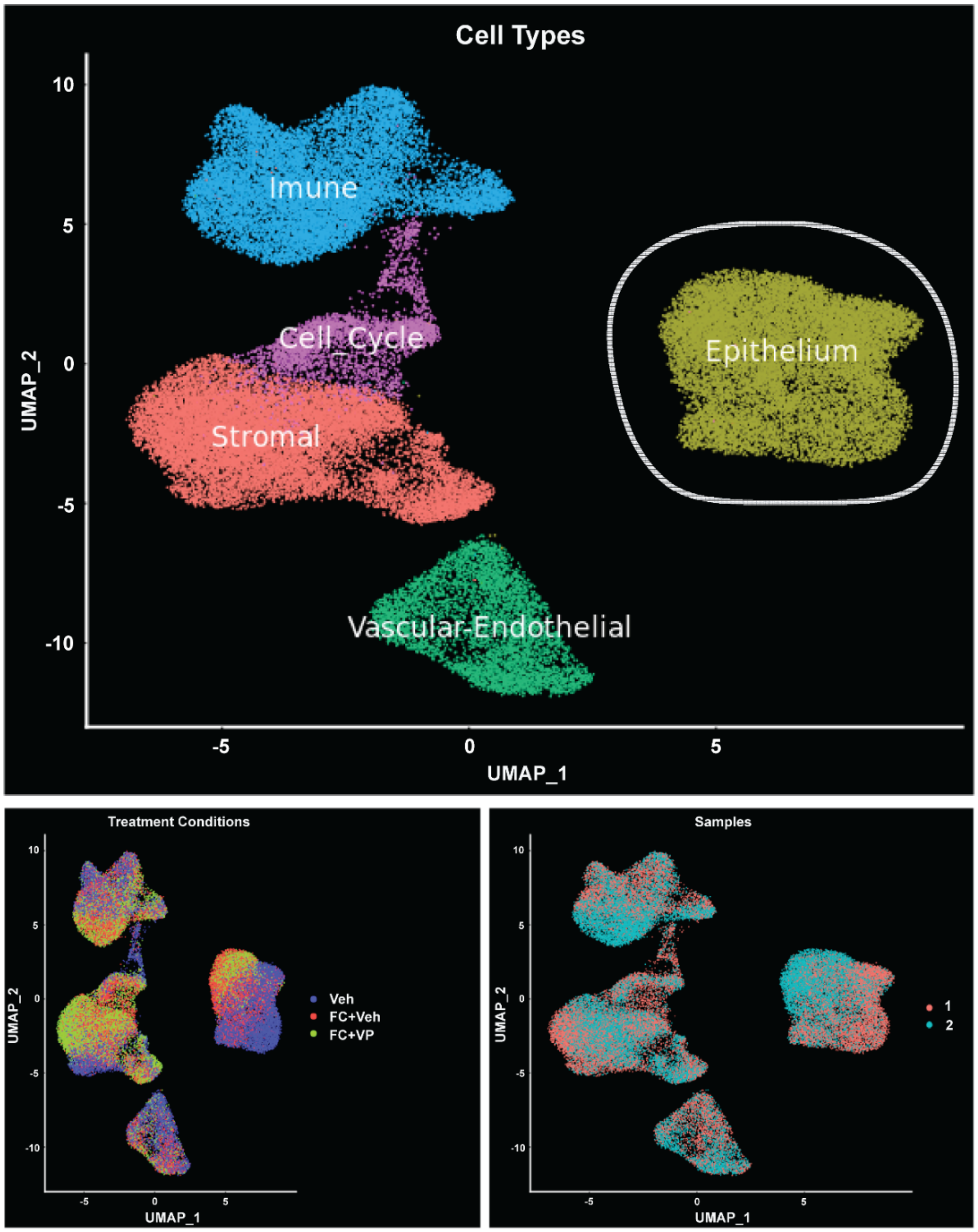

Figure S11. Single Uniform Manifold Approximation and Projection (UMAP) representation of 76259 nuclei from 24 Precision Cut Lung slices generated from 2 control donor lungs (4 PCLS per subject and per condition); each dot represents a single nucleus, and nuclei are labeled by compartment (upper panel) or by condition (4 PCLS per subject and per condition, left lower panel): Veh, FC+Veh, FC+VP and by samples. Source data are provided at doi://10.5281/zenodo.14229565

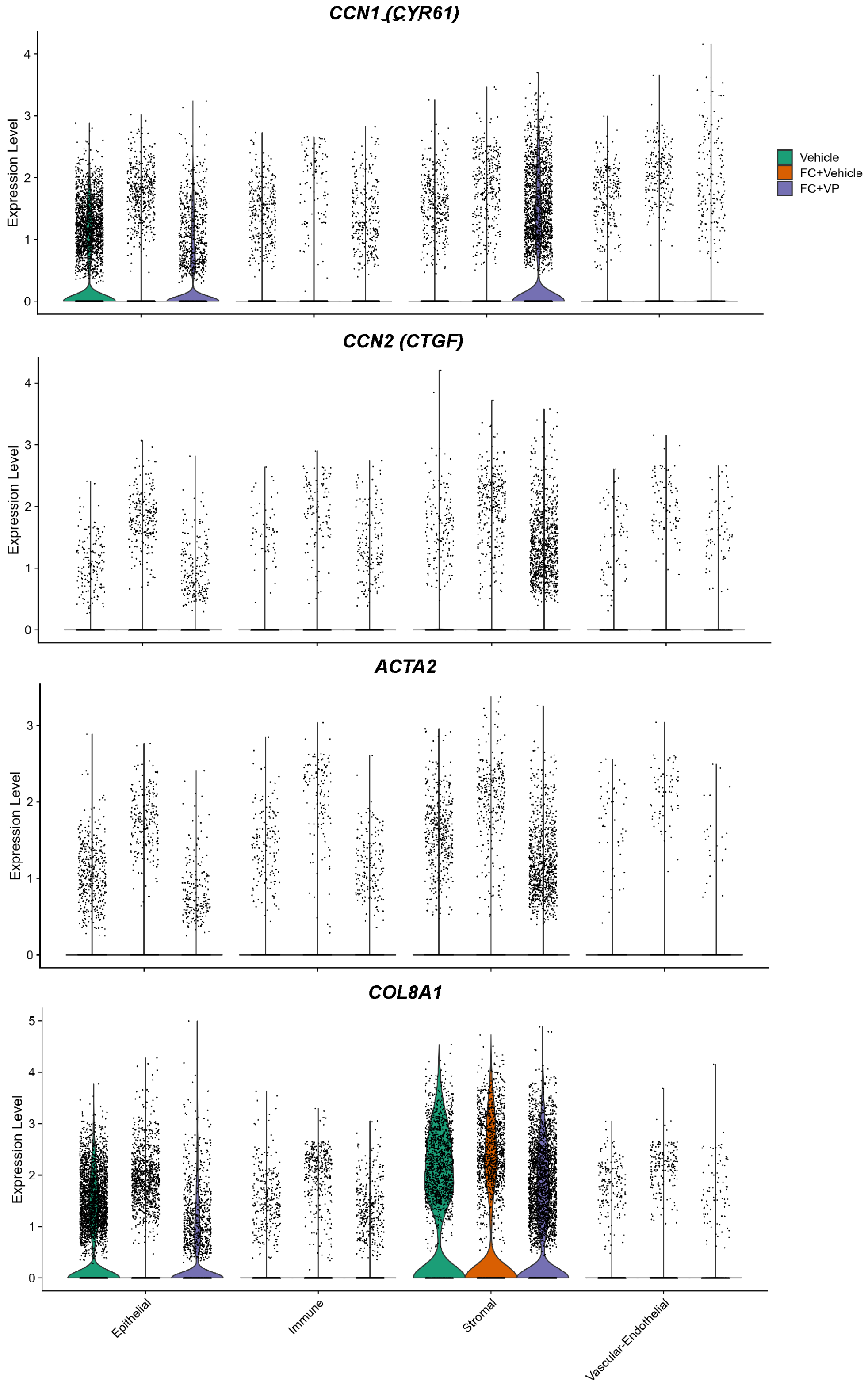

Figure S12. Effects of FC and FC+VP on major cell types. YAP-TEAD targets *CCN1 (CYR61)* and *CCN2 (CTGF)* as well as the classic myofibroblast marker *ACTA2* and the fibrosis-specific fibroblast marker *COL8A1*[4,5]. Source are provided at doi://10.5281/zenodo.14229565

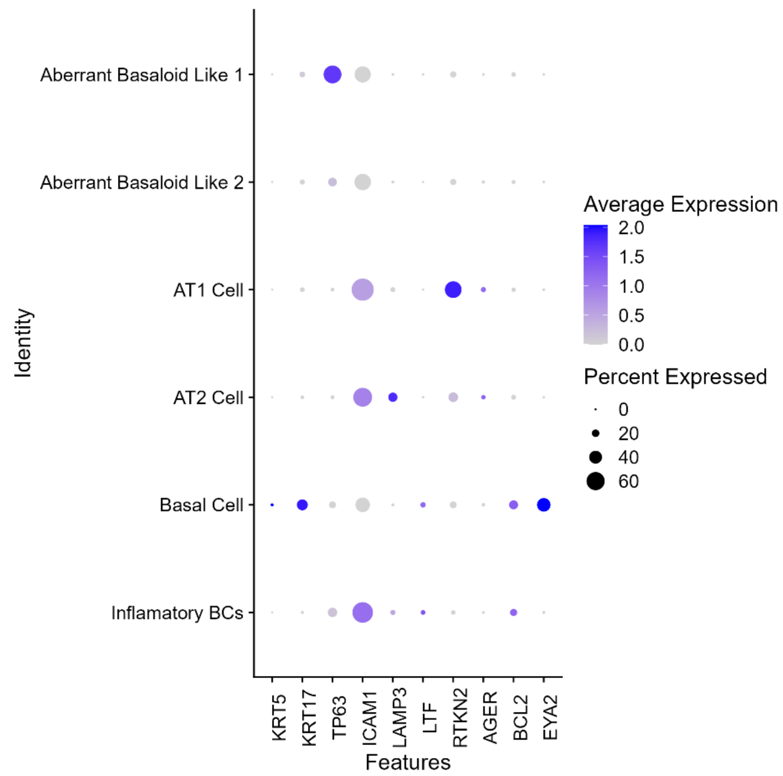

Figure S13. Dot plots corresponding to snRNA-seq data for cluster assignment. Source data are provided at doi://10.5281/zenodo.14229565

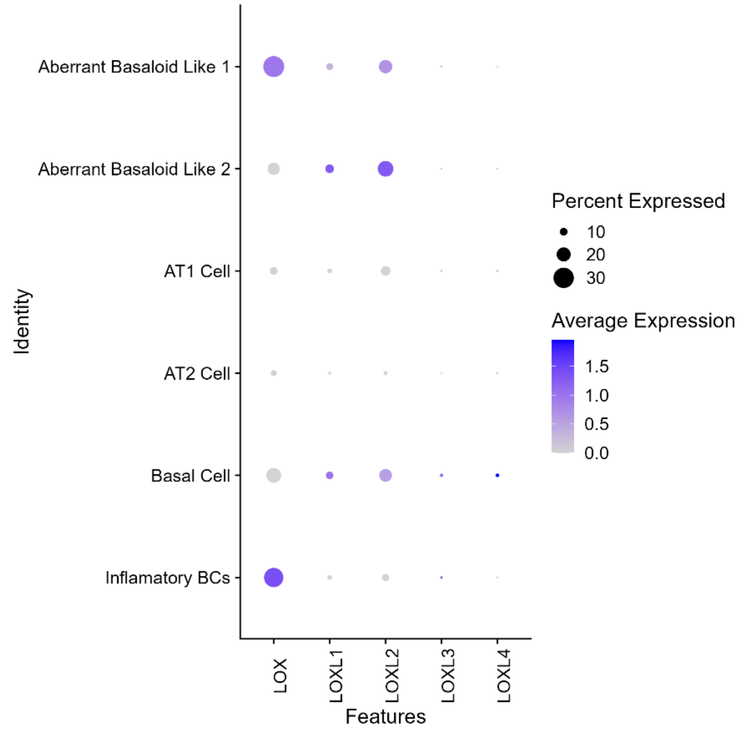

Figures S14. Dot plots of LOX family members corresponding to Figure 6H. Source data are provided at doi://10.5281/zenodo.14229565

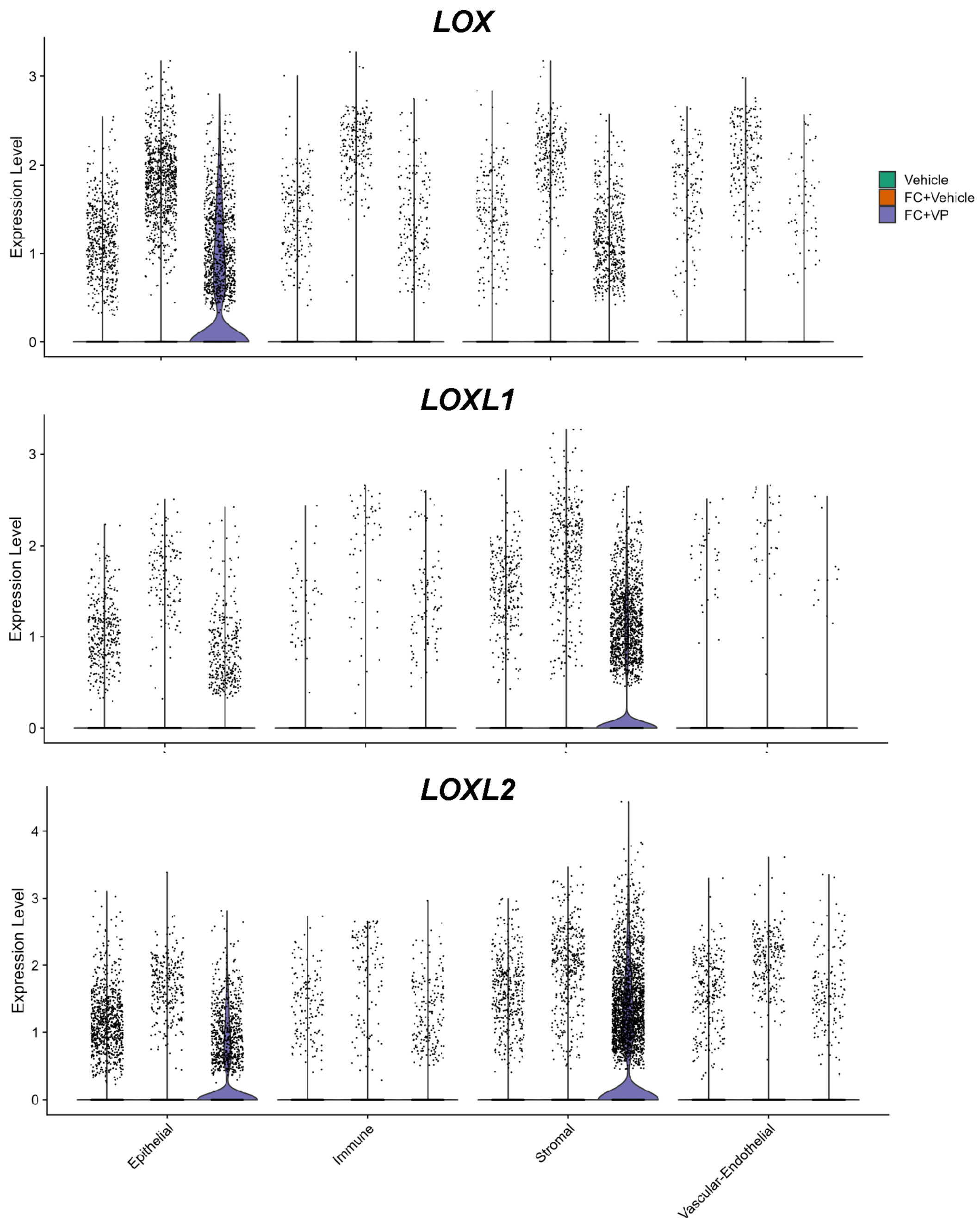

Figure S15. ***Lox, Loxl1*** **and** ***Loxl2*** **expression in all major cell types.** Source data are provided at doi://10.5281/zenodo.14229565

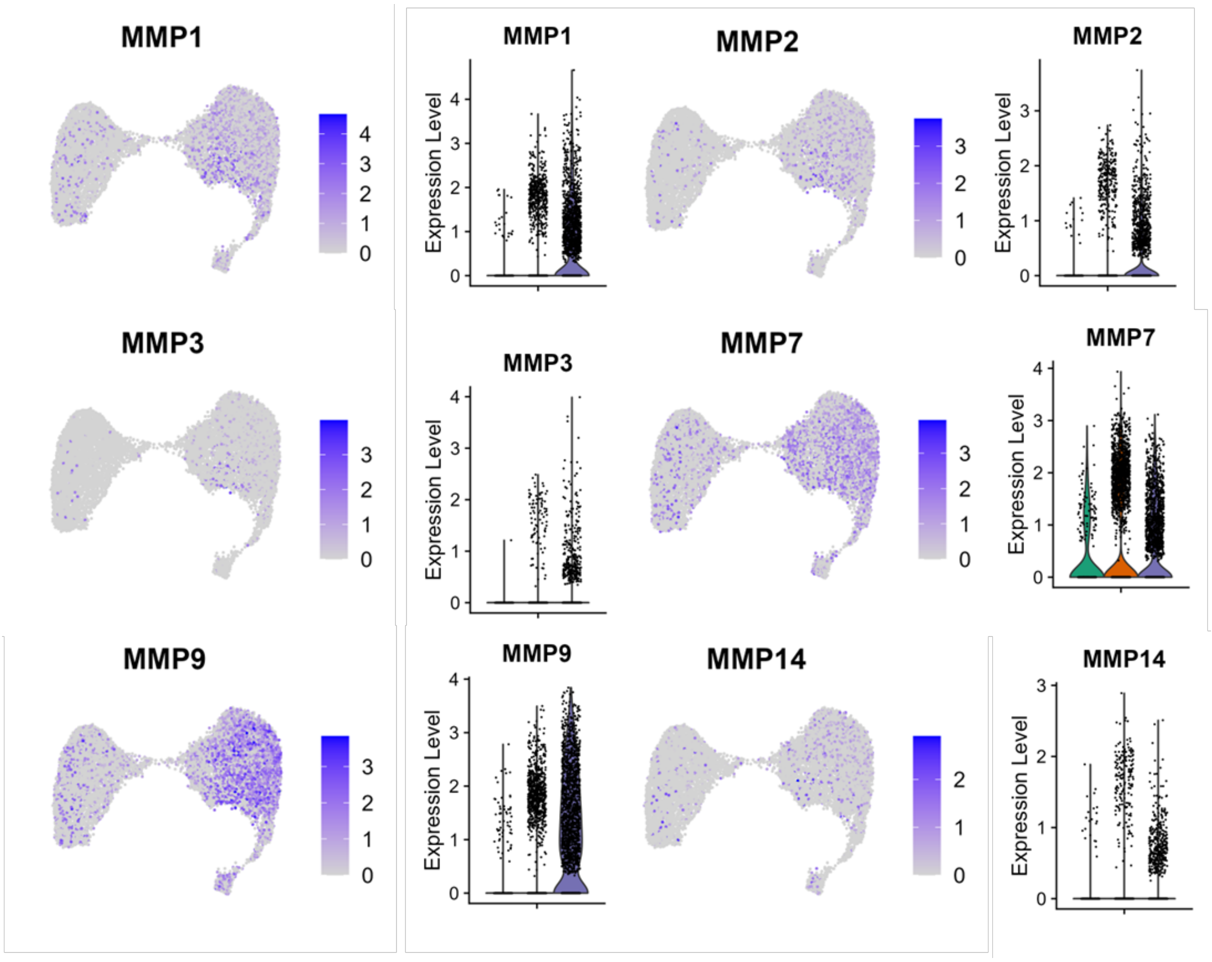

Figure S16. Overview of the most abundantly expressed MMPs in epithelial cells in PCLS treated with FC and verteporfin. The majority of MMPs are elevated with FC treatment but are reduced following VP treatment. From left to right for violin plots: CC, FC, FC+VP. Source data are provided at doi://10.5281/zenodo.14229565

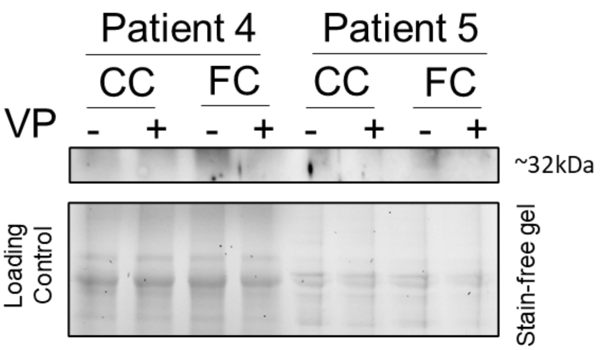

Figure S17. Secreted LOX expression in supernatants collected from 4mm diameter hPCLS treated with CC/FC or verteporfin (FC). Corresponds to Figure 7C-D. Stain-free is shown for qualitative purposes only as it stains tryptophan amino acids only. As collagen does not contain tryptophans, it cannot be accounted for with stain-free technology. Previous work by us[6] and others[7] has shown the FC treatment significantly increases collagen secretion. Therefore, normalization of secreted LOX to the total proteins visualized by the stain-free technology has not been performed here or in Figure 7C-D. Source data are provided as a Source Data file.

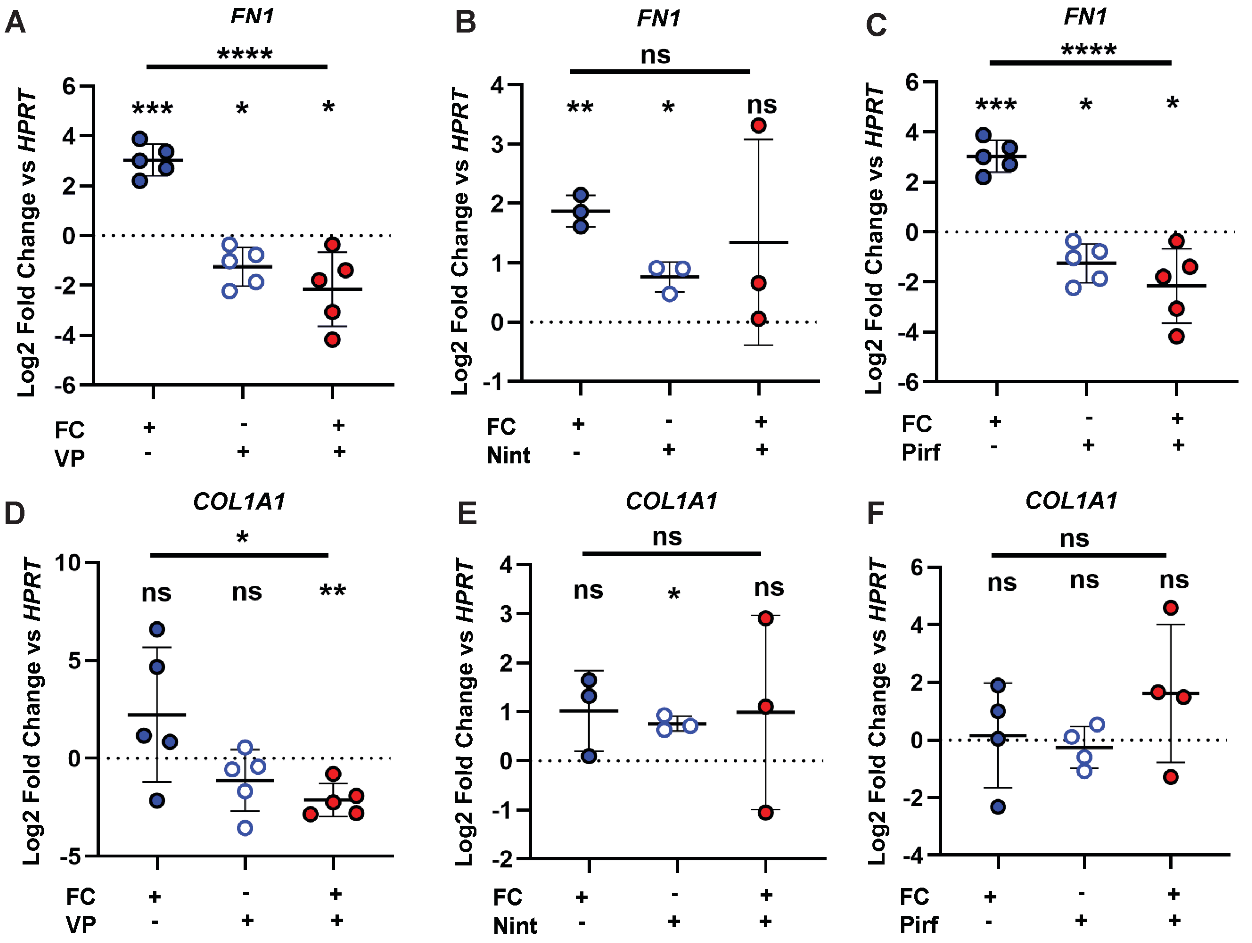

Figure S18. (A-F) Gene expression of *FN1*, and *COL1A1* in PCLS treated with the fibrosis cocktail and VP, Pirfenidone, or Nintedanib. n=3-5 for each drug as indicated in each panel. All data shown as mean ± s.d. Lower statistical row (without lines) is a two-tailed, one sample t-test against a hypothetical value of 0 whereas statistical comparisons between groups and containing lines are One-Way ANOVA with Tukey's multiple comparisons test *p<0.05 considered significant. Source data and statistical analysis for all comparisons are provided as a Source Data file.

Supplementary Tables

Table 1: Hippo pathway gene list used for Principal component analysis (PCA) shown in Figure 1D.

**Hippo Pathway Gene List**	
SAV1	Salvador Family WW Domain Containing Protein 1
AMOT	Angiomotin
YAP1	Yes1 Associated Transcriptional Regulator
YWHAB	Tyrosine 3-Monooxygenase/Tryptophan 5-Monooxygenase Activation Protein Beta
DVL2	Dishevelled Segment Polarity Protein 2
LATS1	Large Tumor Suppressor Kinase 1
WWTR1* (TAZ*)	WW Domain Containing Transcription Regulator 1 or Transcriptional Coactivator With PDZ-Binding Motif
***STK4* (MST1)**	Serine/Threonine Kinase 4 or Mammalian STE20-Like Protein Kinase 1
LATS2	Large Tumor Suppressor Kinase 2
TJP1	Tight Junction Protein 1
AMOTL2	Angiomotin Like 2
TJP2	Tight Junction Protein 2
AMOTL1	Angiomotin Like 1
WWC1	WW And C2 Domain Containing 1
WWC3	WWC Family Member 3

Supplementary Table 2: REACTOME analysis for genes downregulated following siYap/Taz in fibrotic alveolar cells. Corresponds to Figure 3 and S4. P-value adjusted for multiple comparisons using False Discovery Rate

**Pathway name**	**#Entities found**	**#Entities total**	**Entities pValue**	**Submitted entities found**
**YAP1- and WWTR1 (TAZ)-stimulated gene expression**	5	18	1.2955E-05	*Ctgf; Yap1; Kat2b; Wwtr1*
**Signaling by Receptor Tyrosine Kinases**	22	634	0.000687974	*Yap1; Tiam1; Col11a1; Bdnf; Cav1; Ptprz1; Pdgfd; Col4a5; Nrg1; Spry1; Ahcyl1; Mycn; Ap2a2; Gabrb1; Fgf9; Pik3cb; Pcsk6; Vegfd; Met; Pag1*
**Constitutive Signaling by Aberrant PI3K in Cancer**	7	104	0.001646161	*Bdnf; Fgf9; Cd28; Nrg1; Pik3cb; Met*
**PI5P, PP2A and IER3 Regulate PI3K/AKT Signaling**	8	137	0.001899736	*Bdnf; Fgf9; Cd28; Nrg1; Pik3cb; Met; Myd88*
**Negative regulation of the PI3K/AKT network**	8	145	0.002683624	*Bdnf; Fgf9; Cd28; Nrg1; Pik3cb; Met; Myd88*

**Signal Transduction**	67	3049	0.003775269	*Tiam1; Lats2; Aldh1a1; Bdnf; Igf2bp1; Ptprz1; Npy1r; Swap70; Mras; Mycn; Fgf9; Arhgef10; Hist1h3g; Cd28; Camkk2; Pard6b; Edn1; Mmp16; Amotl2; Nrg1; Ap2a2; Rasgrp3; Avpr1a; Ror1; Met; Yap1; Syde1; Smpd3; Syde2; Cxcl10; Col11a1; Pdgfd; Seh1l; Fgd3; Ahcyl1; Ccl7; Gnaq; Nhs; Pik3cb; Vegfd; Pag1; St3gal6; Kat2b; Cav1; Fzd6; Cysltr1; Ackr3; Chn2; Col4a5; Spry1; Myd88; Abca1; Wnt2b; Wwtr1; Rapgef3; Gabrb1; Dlc1; Stard13; Basp1; Pcsk6*
**Assembly of collagen fibrils and other multimeric structures**	5	67	0.004904424	*Ctss; Col11a1; Lox; Col4a5; Col8a1*
**PI3K/AKT Signaling in Cancer**	7	132	0.00600502	*Bdnf; Fgf9; Cd28; Nrg1; Pik3cb; Met*
**Transcriptional Regulation by MECP2**	6	100	0.006037343	*Bdnf; Slc2a3; Met*
**Kidney development**	5	75	0.007780045	*Bmp4; Emx2; Osr1; Slit2*
**Extracellular matrix organization**	12	350	0.012122806	*Bmp4; Efemp1; Ctss; Musk; Col11a1; Lox; Adamts5; Mmp16; Col4a5; Col8a1; Nid2; Fbln5*
**RAC1 GTPase cycle**	8	191	0.013074503	*Swap70; Tiam1; Syde2; Arhgef10; Cav1; Dlc1; Nhs; Chn2*
**CDC42 GTPase cycle**	7	159	0.015433226	*Syde1; Tiam1; Fgd3; Arhgef10; Cav1; Dlc1; Stard13*
**RAC2 GTPase cycle**	5	92	0.017397023	*Syde1; Swap70; Tiam1; Cav1; Nhs*
**Regulation of Insulin-like Growth Factor (IGF) transport and uptake by Insulin-like Growth Factor Binding Proteins (IGFBPs)**	6	127	0.017804546	*Igfbp3; Bmp4; Igfbp2; Prss23; Csf1; Cyr61*
**RAC3 GTPase cycle**	5	100	0.023849531	*Syde1; Swap70; Tiam1; Cav1; Nhs*
**Collagen formation**	5	104	0.027585394	*Ctss; Col11a1; Lox; Col4a5; Col8a1*
**Transcriptional regulation by RUNX3**	5	108	0.03167415	*Ctgf; Yap1; Kat2b; Wwtr1*

**Post-translational protein phosphorylation**	5	109	0.032752435	*Igfbp3; Bmp4; Prss23; Csf1; Cyr61*
**Degradation of the extracellular matrix**	6	148	0.033976134	*Ctss; Col11a1; Adamts5; Mmp16; Col4a5; Col8a1*
**RHO GTPase cycle**	13	460	0.03710145	*Tiam1; Pard6b; Syde1; Syde2; Cav1; Chn2; Swap70; Fgd3; Arhgef10; Dlc1; Stard13; Nhs; Basp1*

Supplementary Table 3: Identifiable Peaks Identified in Paraffin Embedded Lung Tissue Slices Corresponding to IR Bands Found in the Literature

**Position (cm−1)**	**Assignment**	**Description in the literature**	**Ref**
1550	amide II	Collagen	[8-13]
1650	amide I	Collagen trimer (i.e. fibril)	[8,11,13-18]
1662	pyridinoline crosslink	Lox-induced crosslinks	[19]
3080	amide B	Collagen	[10,20,21]
3325	amide A	Collagen	[10,20,21]

Supplementary Table 4. Human primer sets used for RT-qPCR (listed 5' to 3')

**Target Gene**	**Forward Primer**	**Reverse Primer**
CTGF	CTTGCGAAGCTGACCTGGAAGA	CCGTCGGTACATACTCCACAGA
FN1	CCGACCAGAAGTTTGGGTTCT	CAATGCGGTACATGACCCCT
ECAD	ACAGCCCCGCCTTATGATT	CTTCGGAACCGCTTCCTTCA
SFTPC	GCCCAGTGCACCTGAAACGC	TCTCCAGAACCATCTCCGTGTGT
WISP1	TCCAGGCATGAGGTGGTTCC	CAGAATTGGGGGCGTGAGGA
LOX	GCACTCCGATCCTGCTGAT	TGCCCTGTATGCTGTACTGGC
HPRT	AAGGACCCCACGAAGTGTTG	GGCTTTGTATTTTGCTTTTCCA

Supplementary Table 5. Mouse primer sets used for RT-qPCR (listed 5' to 3')

**Target Gene**	**Forward primer**	**Reverse Primer**
Acta2	GCTGGTGATGATGCTCCCA	GCCCATTCCAACCATTACTCC
Ctgf	CTTCTGCGATTTCGGCTCC	TGCTTTGGAAGGACTCACCG
Hprt	CCTAAGATGAGCGCAAGTTGAA	CCACAGGACTAGAACACCTGCTAA
Lox	GTCACCAACATTACCACAGCATGG	GCCTTCAGCCACTCTCCTCTGT
Serpine1	AGGTCAGGATCGAGGTAAACGAG	GGATCGGTCTATAACCATCTCCGT
Wisp1	GTCCTGAGGGTGGGCAACAT	GGGCGTGTAGTCGTTTCCTCT
Loxl1	ACTTTCTCCCCAACCGGCCA	CCTTGTGTCCCTCGGCTACCTT
Col1a1	CCAAGAAGACATCCCTGAAGTCA	TGCACGTCATCGCACACA
Col3a1	GGGGTCCTAGTGGCTTCCGA	GCTCTCCGGGAGGACCCTTT

Supplementary Table 6. Antibodies used for immunohistochemistry (IHC), immunofluorescence (IF) and western blot (WB)

Target	Product No.	Company	Dilutions
YAP1 (EP1674Y) (IHC - h)	ab52771	abcam	1:75
TAZ (IHC - h)	ab 84927	abcam	1:75
KRT5 (IHC -h)	ab75869	abcam	1:200
α-SMA (IHC – h)	ab5694	abcam	1:100
KRT7 (IHC – h)	ab68459	abcam	1:200
HOPX (IHC – h)	ab230544	abcam	1:200
Pro-SPC (IHC – h)	ab3786	Millipore	1:800
YAP/TAZ D24E4 (WB, IF – m)	#8418	Cell Signaling	1:200, WB-1:1000
LOX (WB, IF – h, m)	ab31238	abcam	WB-1:200; IF-1:100
beta-actin (m)	A3854	Sigma-Aldrich	1:50000
E-CAD (IF, m)	BD610182	BD Biosciences	1:200
Collagen 1 (WB, m)	600-401-103	Rockland	1:250
YAP (IF- h)	sc-376830	Santa Cruz	1:100
LOX (IF -h)	NB100-2527	Novus Biologicals	1:100
HTII-280 (IF -h)	TB-27AHT2-280	Terrace Biotech	1:300
DC-LAMP (IF-m)	DDX0191P-100	Novus Biologicals	1:100
Cy5 (secondary)	20811	Biotium	1:2000
Cy7 (secondary)	20463	Biotium	1:2000

References

1 Alsafadi, H. N. *et al.* Simultaneous isolation of proximal and distal lung progenitor cells from individual mice using a 3D printed guide reduces proximal cell contamination of distal lung epithelial cell isolations. *Stem Cell Reports* **17**, 2718-2731, doi:https://doi.org/10.1016/j.stemcr.2022.11.002 (2022).

2 Heberle, H., Meirelles, G. V., da Silva, F. R., Telles, G. P. & Minghim, R. InteractiVenn: a web-based tool for the analysis of sets through Venn diagrams. *BMC Bioinformatics* **16**, 169, doi:10.1186/s12859-015-0611-3 (2015).

3 Wershof, E. *et al.* A FIJI macro for quantifying pattern in extracellular matrix. *Life Science Alliance* **4**, e202000880, doi:10.26508/lsa.202000880 (2021).

4 Jia, M. *et al.* Early events marking lung fibroblast transition to profibrotic state in idiopathic pulmonary fibrosis. *Respir Res* **24**, 116, doi:10.1186/s12931-023-02419-0 (2023).

5 Xie, T. *et al.* Single-Cell Deconvolution of Fibroblast Heterogeneity in Mouse Pulmonary Fibrosis. *Cell Reports* **22**, 3625-3640, doi:https://doi.org/10.1016/j.celrep.2018.03.010 (2018).

6 Alsafadi, H. N. *et al.* An ex vivo model to induce early fibrosis-like changes in human precision-cut lung slices. *American Journal of Physiology-Lung Cellular and Molecular Physiology* **312**, L896-L902, doi:10.1152/ajplung.00084.2017 (2017).

7 Machahua, C., Marti, T. M., Dorn, P. & Funke-Chambour, M. Fibrosis in PCLS: comparing TGF-β and fibrotic cocktail. *Respir Res* **26**, 44, doi:10.1186/s12931-025-03110-2 (2025).

8 Camacho, N. P., West, P., Torzilli, P. A. & Mendelsohn, R. FTIR microscopic imaging of collagen and proteoglycan in bovine cartilage. *Biopolymers* **62**, 1-8, doi:10.1002/1097-0282(2001)62:1<1::AID-BIP10>3.0.CO;2-O (2001).

9 Le Cerf, B. A. *et al.* Nano-Mechanical Analyses of Native and Cross-Linked Collagen I Matrices Reveal the Mechanical Complexity of Homogenous Samples. *Front Phys-Lausanne* **10**, doi:10.3389/fphy.2022.835038 (2022).

10 Martinez Cortizas, A. & Lopez-Costas, O. Linking structural and compositional changes in archaeological human bone collagen: an FTIR-ATR approach. *Sci Rep* **10**, 17888, doi:10.1038/s41598-020-74993-y (2020).
11 Nashchekina, Y. A. Molecular and fibrillar structure collagen analysis by FTIR spectroscopy. *Journal of Physics: Conference Series*, doi:10.1088/1742-6596/1697/1/012053 (2020).
12 Fraser, R. D. B. M. T. P. *Conformation in Fibrous Proteins and Related Synthetic Polypeptides*. (1973).
13 de Campos Vidal, B. & Mello, M. L. S. Collagen type I amide I band infrared spectroscopy. *Micron* **42**, 283-289, doi:https://doi.org/10.1016/j.micron.2010.09.010 (2011).
14 Belbachir, K., Noreen, R., Gouspillou, G. & Petibois, C. Collagen types analysis and differentiation by FTIR spectroscopy. *Anal Bioanal Chem* **395**, 829-837, doi:10.1007/s00216-009-3019-y (2009).
15 Jastrzebska, M. *et al.* Atomic force microscopy and FT-IR spectroscopy investigations of human heart valves. *Gen Physiol Biophys* **25**, 231-244 (2006).
16 Lazarev, Y. A., Grishkovsky, B. A. & Khromova, T. B. Amide I band of IR spectrum and structure of collagen and related polypeptides. *Biopolymers* **24**, 1449-1478, doi:10.1002/bip.360240804 (1985).
17 Tiong, W. H., Damodaran, G., Naik, H., Kelly, J. L. & Pandit, A. Enhancing amine terminals in an amine-deprived collagen matrix. *Langmuir* **24**, 11752-11761, doi:10.1021/la801913c (2008).
18 Sellaro, T. L. *et al.* Effects of collagen fiber orientation on the response of biologically derived soft tissue biomaterials to cyclic loading. *J Biomed Mater Res A* **80**, 194-205, doi:10.1002/jbm.a.30871 (2007).
19 Mieczkowska, A. & Mabilleau, G. Validation of Fourier Transform Infrared Microspectroscopy for the Evaluation of Enzymatic Cross-Linking of Bone Collagen. *Calcified Tissue International* **113**, 344-353, doi:10.1007/s00223-023-01105-z (2023).
20 Li, J., Li, Y., Li, Y., Yang, Z. & Jin, H. Physicochemical Properties of Collagen from Acaudina Molpadioides and Its Protective Effects against H(2)O(2)-Induced Injury in RAW264.7 Cells. *Mar Drugs* **18**, doi:10.3390/md18070370 (2020).
21 Liu, S. Investigation of the solubility and dispersion degree of calf skin collagen in ionic liquids (vol 1, 11, 2019). *Collagen Leather* **5** (2024).
