# Supplementary material for: Inhibition of epithelial cell YAP-TEAD/LOX signaling attenuates pulmonary fibrosis in preclinical models
Source: Nat Commun. 2025 Aug 2;16:7099. doi: 10.1038/s41467-025-61795-x (PMC12318044; doi:10.1038/s41467-025-61795-x)
Supplement: Supplementary file 2 — Reporting Summary [file 41467_2025_61795_MOESM2_ESM.pdf]

nature portfolio

Corresponding author(s): Melanie Koenigshoff

Last updated by author(s): 2025--1-16

Reporting Summary

Nature Portfolio wishes to improve the reproducibility of the work that we publish. This form provides structure for consistency and transparency in reporting. For further information on Nature Portfolio policies, see our Editorial Policies and the Editorial Policy Checklist.

Statistics

For all statistical analyses, confirm that the following items are present in the figure legend, table legend, main text, or Methods section.

n/a	Confirmed	
☐	☒	The exact sample size (*n*) for each experimental group/condition, given as a discrete number and unit of measurement
☐	☒	A statement on whether measurements were taken from distinct samples or whether the same sample was measured repeatedly
☐	☒	The statistical test(s) used AND whether they are one- or two-sided<br>*Only common tests should be described solely by name; describe more complex techniques in the Methods section.*
☐	☒	A description of all covariates tested
☐	☒	A description of any assumptions or corrections, such as tests of normality and adjustment for multiple comparisons
☐	☒	A full description of the statistical parameters including central tendency (e.g. means) or other basic estimates (e.g. regression coefficient) AND variation (e.g. standard deviation) or associated estimates of uncertainty (e.g. confidence intervals)
☐	☒	For null hypothesis testing, the test statistic (e.g. *F*, *t*, *r*) with confidence intervals, effect sizes, degrees of freedom and *P* value noted<br>*Give P values as exact values whenever suitable.*
☒	☐	For Bayesian analysis, information on the choice of priors and Markov chain Monte Carlo settings
☒	☐	For hierarchical and complex designs, identification of the appropriate level for tests and full reporting of outcomes
☐	☒	Estimates of effect sizes (e.g. Cohen's *d*, Pearson's *r*), indicating how they were calculated

Our web collection on statistics for biologists contains articles on many of the points above.

Software and code

Policy information about availability of computer code

Data collection	Data collected from RNA samples from mouse and human tissue and cells. RNA quality control was performed using High Sensitivity RNA ScreenTape for an Agilent TapeStation. Transcriptomic analysis was performed using Clariom™ D mouse microarrays.<br><br>Final snRNAseq libraries were analyzed on an Agilent Bioanalyzer High Sensitivity DNA chip for qualitative control purposes. cDNA libraries were sequenced on a HiSeq 4000 Illumina platform aiming for 150 million reads per library and a sequencing configuration of 26 base pair (bp) on read1 and 98 bp on read2.<br><br>Code used to generate the various figures in this manuscript can be found on the github repository:https://github.com/Lung-bioengineering-regeneration-lab/Hippo_LOX. Other softwares used were as follows: NDP.view2 software for immunohistochemistry, Olympus OlyVia 3.1 Software for immunofluorescence of human lung tissue, murine Masson's trichrome stain: MIRAX Scan (Zeiss) with Control Software v0.5. Protein bands were visualized using Biorad's ChemiDOC XPS (Biorad). Gelation assays were conducted using a Cytation5 and Gen5 Software. 5um lung tissue slices from paraffin embedded samples were analyzed in transmission mode using a Diamond-ATR accessory for a Cary630 FTIR with KBr optics (Agilent).
Data analysis	Raw data from microarrays performed on normal and fibrotic AT2 cells treated with or without siRNA for Yap/Taz are deposited in the ArrayExpress under accession number E-MTAB-14643.<br><br>10X Genomics data and metadata generated for this study are available in Zenodo database (10.5281/zenodo.14229565).

Basecalls were converted to reads with the software Cell Ranger's (v4.0.0) implementation mkfastq.
MTC stained and scanned murine lungs (MIRAX Viewer Software v.1.12.25.3)
Immunofluorescence staining: Axio Imager M2 Microscope (Zeiss) or confocal microscope (LSM 710; Zeiss).

Protein bands were quantified using ImageLab v6.0 (Biorad)

Paraffin spectra were collected and subtracted from all samples. Spectra were smoothened using a Savitzky-Golay filter with order 3 and length 51 prior to background subtraction in R. Quasar version 1.10.2, an open-source toolbox extending the capabilities of Orange 3.37.0 and Orange Spectroscopy v.0.7.2

For manuscripts utilizing custom algorithms or software that are central to the research but not yet described in published literature, software must be made available to editors and reviewers. We strongly encourage code deposition in a community repository (e.g. GitHub). See the Nature Portfolio guidelines for submitting code & software for further information.

Data

Policy information about availability of data

All manuscripts must include a data availability statement. This statement should provide the following information, where applicable:

- Accession codes, unique identifiers, or web links for publicly available datasets
- A description of any restrictions on data availability
- For clinical datasets or third party data, please ensure that the statement adheres to our policy

The raw data from microarrays performed on normal and fibrotic AT2 cells treated with or without siRNA for Yap/Taz have been deposited in the ArrayExpress database under accession number E-MTAB-14643.
All murine histological slides used for Modified Ashcroft scoring, human immunohistochemistry of parallel sections of YAP/TAZ with co-stains, and SEM images of collagen networks for quantification have been deposited in the BioImage Archive S-BIAD1520. 10X Genomics data and metadata generated for this study have been deposited in the Zenodo database (https://doi.org/10.5281/zenodo.14229565). All other data generated in this study are provided in the Supplementary Information/Source Data file.
Source data are provided with this paper.

Research involving human participants, their data, or biological material

Policy information about studies with human participants or human data. See also policy information about sex, gender (identity/presentation), and sexual orientation and race, ethnicity and racism.

Reporting on sex and gender	we report on sex
Reporting on race, ethnicity, or other socially relevant groupings	we report on race and ethnicity, as available for our human cohorts.
Population characteristics	Fresh lung tissue of explanted Donor or IPF lungs.
Recruitment	Tissue from deceased individuals.
Ethics oversight	The study was approved by the local ethics committee of the Ludwig-<br>606 Maximilians University of Munich, Germany (Ethic vote #19-630) and University of Pittsburgh<br>607 (IRB PRO14010265). Written informed consent was obtained for all study participants

Note that full information on the approval of the study protocol must also be provided in the manuscript.

Field-specific reporting

Please select the one below that is the best fit for your research. If you are not sure, read the appropriate sections before making your selection.

☒ Life sciences ☐ Behavioural & social sciences ☐ Ecological, evolutionary & environmental sciences

For a reference copy of the document with all sections, see nature.com/documents/nr-reporting-summary-flat.pdf

Life sciences study design

All studies must disclose on these points even when the disclosure is negative.

Sample size	Animal experiments have been conducted following the ARRIVE guidelines. The number of mice used for the studies was mainly based on the historical use of specific time points of the bleomycin mouse model, which allows us to detect a 20% change in lung function and hydroxyproline. Samples sizes for in vivo and primary alveolar type II cell isolations were determined for all experiments based on power calculations performed using previous data in the lab.<br>Most of the differences that we observed were statistically significant, demonstrating the suitability of the sample size outlined in the

mansucript

Data exclusions

For the single-nuclear RNAseq data, cells were filtered for quality control. Otherwise, no data were excluded.

Replication

The results reported in this manuscript were reproducible in independent experiments as stated in the legends, If results from insufficient numbers for statistical analysis, are shown, this limitation is explicilty mentioned.

Randomization

Animals were allocated into groups prior to the onset of any experimentation (i.e. upon arrival to the facility when placed in cages). For PCLS generation, slices were generated and randomly allocated into a 96 well plate with a pre-determined layout for groupings.

Blinding

Blinding to group allocation was done for all animal experimentation for those collecting data (i.e. qPCR data) and was unblinded for analysis as group allocation needed to be identified at that time. Blinding was not possible for immunofluorescence or western blot due to the fact that specific regions of interest needed to be identified and for western blot, samples needed to be loaded in a certain order for the generation of publishable blots.
For PCLS experiments, due to the highly specialized nature of the work, it was not possible to blind the person performing treatment and analysis.

Reporting for specific materials, systems and methods

We require information from authors about some types of materials, experimental systems and methods used in many studies. Here, indicate whether each material, system or method listed is relevant to your study. If you are not sure if a list item applies to your research, read the appropriate section before selecting a response.

Materials & experimental systems

n/a	Involved in the study	
☐	☒	Antibodies
☒	☐	Eukaryotic cell lines
☒	☐	Palaeontology and archaeology
☐	☒	Animals and other organisms
☒	☐	Clinical data
☒	☐	Dual use research of concern
☒	☐	Plants

Methods

n/a	Involved in the study	
☒	☐	ChIP-seq
☒	☐	Flow cytometry
☒	☐	MRI-based neuroimaging

Antibodies

Antibodies used

YAP1 (IHC - h)
ab52771
abcam
TAZ (IHC - h)
ab 84927
abcam
KRT5 (IHC -h)
ab75869
abcam
α-SMA (IHC – h)
ab5694
abcam
KRT7 (IHC – h)
ab68459
abcam
HOPX (IHC – h)
ab230544
abcam
Pro-SPC (IHC – h)
ab3786
Millipore
YAP/TAZ (WB, IF – m)
D24E4
Cell Signaling
LOX (WB, IF – h, m)
ab31238
abcam
E-CAD (IF, m)
BD610182
BD Biosciences
Collagen 1 (WB, m)
600-401-103

Rockland
YAP (IF- h)
sc-376830
Santa Cruz
LOX (IF -h)
NB100-2527
Novus Biologicals
HTII-280 (IF -h)
TB-27AHT2-280
Terrace Biotech
DC-LAMP (IF-m)
DDX0191P-100
Novus Biologicals
Cy5 (secondary) - 20811 Biotium
Cy7 (secondary) - 20463 Biotium

Validation	All antibodies have been validated by the manufacturer and by multiple citations for reactivity against mouse or human. For immunostaining: all antibodies used in mouse or human tissue have been validated by the manufacturers (i.e. antibodies above) and by multiple citations for mouse or human reactivity and for use in immunofluorescence staining. Where not tested for previous projects in our laboratories, negative controls were performed.

Animals and other research organisms

Policy information about studies involving animals; ARRIVE guidelines recommended for reporting animal research, and Sex and Gender in Research

Laboratory animals	For bleomycin experiments: C57BL/6J mice (all female) were purchased from Charles River Germany and maintained at the appropriate biosafety level at constant temperature and humidity with a 12 hour light cycle. At the age of 9-12 weeks, mice were instilled with bleomycin or PBS.
Wild animals	The study did not involve wild animals.
Reporting on sex	Reports on sex are included. 12-week-old female C57BL/6 mice were used for all animal experiments as indicated.
Field-collected samples	The study did not involve samples collected from the field.
Ethics oversight	The use of animals was approved under the ethics of the Helmholtz-Zentrum Munich and the state of Bavaria, Germany; project number: 55.2-1-54-2532-88-12.

Note that full information on the approval of the study protocol must also be provided in the manuscript.

Plants

Seed stocks	*Report on the source of all seed stocks or other plant material used. If applicable, state the seed stock centre and catalogue number. If plant specimens were collected from the field, describe the collection location, date and sampling procedures.*
Novel plant genotypes	*Describe the methods by which all novel plant genotypes were produced. This includes those generated by transgenic approaches, gene editing, chemical/radiation-based mutagenesis and hybridization. For transgenic lines, describe the transformation method, the number of independent lines analyzed and the generation upon which experiments were performed. For gene-edited lines, describe the editor used, the endogenous sequence targeted for editing, the targeting guide RNA sequence (if applicable) and how the editor was applied.*
Authentication	*Describe any authentication procedures for each seed stock used or novel genotype generated. Describe any experiments used to assess the effect of a mutation and, where applicable, how potential secondary effects (e.g. second site T-DNA insertions, mosiacism, off-target gene editing) were examined.*
